# Supplementary material for: Chromosome Fissions and Fusions Act as Barriers to Gene Flow between Brenthis Fritillary Butterflies
Source: Mol Biol Evol. 2023 Feb 22;40(3):msad043. doi: 10.1093/molbev/msad043 (PMC10015618; doi:10.1093/molbev/msad043)
Supplement: msad043_Supplementary_Data [file msad043_supplementary_data.pdf]

# Supplementary Materials

## Supplementary Note 1

The ability to accurately infer effective demographic parameters from the bSFS depends on a number of variables. A single block only contains information about a single genealogy, so many blocks are required to make accurate inference and statistcal power depends on the amount of recombination between blocks. However, recombination within blocks can introduce bias, because the analytic calculation for the bSFS assumes no recombination within blocks. So recombination involves a trade-off between power and potential for bias in parameter and model estimates.

With this in mind, we used gIMble simulate to investigate how recombination affects our ability to estimate demographic parameters in windows across the genome. We simulated windows of equivalent size as those in the real data under the best fitting genome-wide demographic model (Figure 2C). Each simulation contained 30,030 blocks of 64 bases, equivalent to a window of length 45.76 kb split up into 715 blocks. The bSFS was calculated by recording the mutation counts for all 42 possible pairwise comparisons ($6 \times 7$ unphased diploid individuals from *B. ino* and *B. daphne* respectively).

We performed 100 replicate simulations for six different per-base recombination rates ($r$): $1 \times 10^{-9}$, $5 \times 10^{-9}$, $9 \times 10^{-9}$, $1.3 \times 10^{-8}$, $1.7 \times 10^{-8}$, $2.1 \times 10^{-8}$. We estimated demographic parameters ($N_e$ and $m_e$) for each replicate, while fixing the split time ($T$), analogous to the the window-wise analysis on the real data. However, we used free optimisation rather than a grid, because the former provides finer parameter estimates. Importantly, it is possible to identify simulation replicates which have not converged to their maximum composite lnCL (MCL), because the parameters they were simulated under are known. This task is much more challenging with the *Brenthis* data (because the parameters are unknown) and so requires the use of a grid.

Comparing results under different recombination rates (Figure S3), we find that there is little power to accurately estimate $m_e$, the parameter we are most interested in, when the recombination rate is $1 \times 10^{-9}$. As recombination increases, estimates of $m_e$ become closer to the true value ($1.811 \times 10^{-7}$). However, $m_e$ is often underestimated at higher recombination rates (Figure S3). For example, the mean estimate of $m_e$ when $r = 2.1 \times 10^{-8}$ is $1.195 \times 10^{-7}$.

This power analysis on simulated data shows that given plausible recombination rates, our

analyses of $m_e$ in windows (of 30,000 consecutive blocks) have reasonable power, even though estimates suffer from some downward bias. Although we lack estimates of recombination rate in *B. daphne* and *B. ino*, we expect the mean crossover rate to be approximately $8.5 \times 10^{-9}$ (equivalent to a single crossover per male meiosis for 14 chromosome pairs). In addition, windows in the *Brenthis* dataset often span much greater distances than 45.76 kb (median window span 122 kb) because genic and repetitive regions are removed. This increases the amount of between-block recombination and therefore power.

## Supplementary Note 2

Throughout our analyses we have assumed that the arrangement of chromosomes found in each genome assembly is representative of each species, i.e. rearrangements are fixed between species. Although all chromosome rearrangements we have identified are homozygous in the reference assemblies, it is still possible that a small number of rearrangements are polymorphic within species. A potential consequence of polymorphic rearrangements is that they act as barriers to gene flow within a species. We tested for this possibility by repeating our demographic analysis at the intraspecific level.

We fit genome-wide demographic models to estimate the divergence history of populations that currently occupy different glacial refugia. We inferred that *B. ino* from Iberian and Balkan populations ($F_{\mathrm{ST}} = 0.118$) split approximately 459 kya without post-divergence gene flow (Table S2). By contrast, *B. daphne* Iberian and Balkan populations ($F_{\mathrm{ST}} = 0.112$) likely split more recently (331 kya) and with considerable post-divergence gene flow ($m_e = 1.072 \times 10^{-5}$) from Iberian to the Balkan populations forwards in time (Table S2).

We then estimated variation in $m_e$ between Iberian and Balkan populations of *B. daphne* across the genome. The distribution of $m_e$ estimates on non-rearranged chromosomes, rearranged chromosomes, and within 1 Mb of rearrangement points, are all very similar (Figure S4). There are no statistically significant differences between their means (permutation tests, see Methods). This is in stark contrast to the interspecific results (Figure 4), thus demonstrating that these rearrangements are barriers to gene flow between species but not between refugial populations within *B. daphne*.

Table S1: Sampling locations and other metadata for individuals used in this study. In the data column, the source of the data is denoted as TS (This Study) or M2022 (Mackintosh *et al.* 2022).

| Sample | Date | Species | Sex | Locality | Region | Country | Lat | Long | Collector | Data |
|---|---|---|---|---|---|---|---|---|---|---|
| ES_BD_1141 | 25/7/2018 | daphne | Female | Meranges, La Cerdanya | Catalunya | Spain | 42.435 | 1.797 | RV, Sabina Vila | Pacbio, WGS; TS |
| ES_BD_1489 | 29/7/2009 | daphne | Female | Prioro | Castile and León | Spain | 42.937 | -4.964 | RV | WGS; TS |
| ES_BD_1490 | 24/7/2008 | daphne | Female | Uña | Castile-La Mancha | Spain | 40.231 | -1.960 | RV | WGS; TS |
| FR_BD_1329 | 15/7/2019 | daphne | Female | D620 | Aude | France | 42.997 | 2.053 | KL | WGS, HiC; TS |
| GR_BD_1491 | 26/7/2013 | daphne | Female | Rhodopi, Frakto Forest | Drama | Greece | 41.504 | 24.400 | RV | WGS; TS |
| IT_BD_1493 | 26/6/2012 | daphne | Male | Saguccio, Aspromonte | Aspromonte | Italy | 38.080 | 15.830 | RV | WGS; TS |
| RO_BD_956 | 17/7/2018 | daphne | Male | Pin1000m, Lupsa, Apuseni Mt. | Alba | Romania | 46.416 | 23.192 | KL, AH, DL, RV | WGS; TS |
| ES_BI_364 | 05/07/2017 | ino | Male | Somiedo, Braña de Mumian | Asturias | Spain | 43.068 | -6.24 | KL | WGS, reference genome; M2022 |
| ES_BI_375 | 05/07/2017 | ino | Male | Somiedo, Braña de Mumian | Asturias | Spain | 43.068 | -6.24 | KL | WGS; M2022 |
| FR_BI_1497 | 11/08/2012 | ino | Female | Larche (Les Marmottes) | Alpes-de-Haute-Provence | France | 44.446 | 6.851 | Vlad Dincă, Raluca Vodă | WGS; M2022 |
| RS_BI_1496 | 29/6/2014 | ino | Male | Čeganica | - | Serbia | 43.396 | 22.368 | RV | WGS; TS |
| SE_BI_1495 | 13/7/2016 | ino | Female | Älvsbyn | Norrbotten | Sweden | 65.668 | 20.955 | RV | WGS; TS |
| UA_BI_1494 | 20/6/2014 | ino | Female | Kruglyanka, Novaya Vodolaga | Kharkiv oblast | Ukraine | 49.817 | 35.733 | RV | WGS; TS |

Table S2: Maximum composite likelihood parameters for intraspecific demographic models. Parameter estimates and log composite likelihoods (lnCL) are shown for $IM$ models of divergence between Iberian and Balkan populations of *B. ino* and *B. daphne*. The $N_e$ and split time parameter estimates are in units of $10^6$ individuals and years, respectively.

| **Species** | **Model** | $N_e$ ***Balkans*** | $N_e$ ***Iberia*** | $N_e$ ***ancestral*** | $m_e$ | **Split time** | **lnCL** |
|---|---|---|---|---|---|---|---|
| *ino* | $IM_{\to Balk}$ | 1.025 | 0.700 | 1.019 | $5.949 \times 10^{-20}$ | 0.459 | -22,993,925 |
| *ino* | $IM_{\to Iber}$ | 1.025 | 0.700 | 1.019 | 0 | 0.459 | -22,993,925 |
| *daphne* | $IM_{\to Balk}$ | 0.101 | 0.102 | 1.090 | $1.072 \times 10^{-5}$ | 0.331 | -17,408,294 |
| *daphne* | $IM_{\to Iber}$ | 0.222 | 0.012 | 1.239 | $3.510 \times 10^{-5}$ | 0.646 | -17,422,413 |

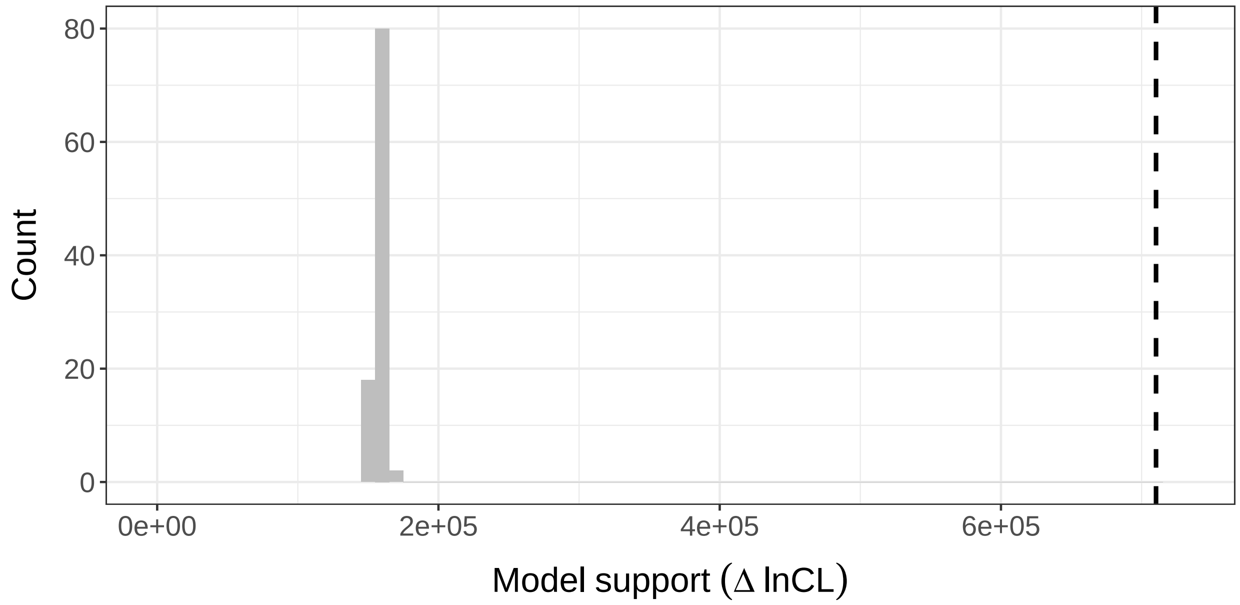


Figure S1: A histogram showing the improvement in model support (Δ lnCL) between the $DIV$ and $IM_{\rightarrow Bda}$ models for 100 parametric bootstrap replicates, each simulated under the same $DIV$ history. The improvement in fit for the real data is marked with a dashed vertical line.

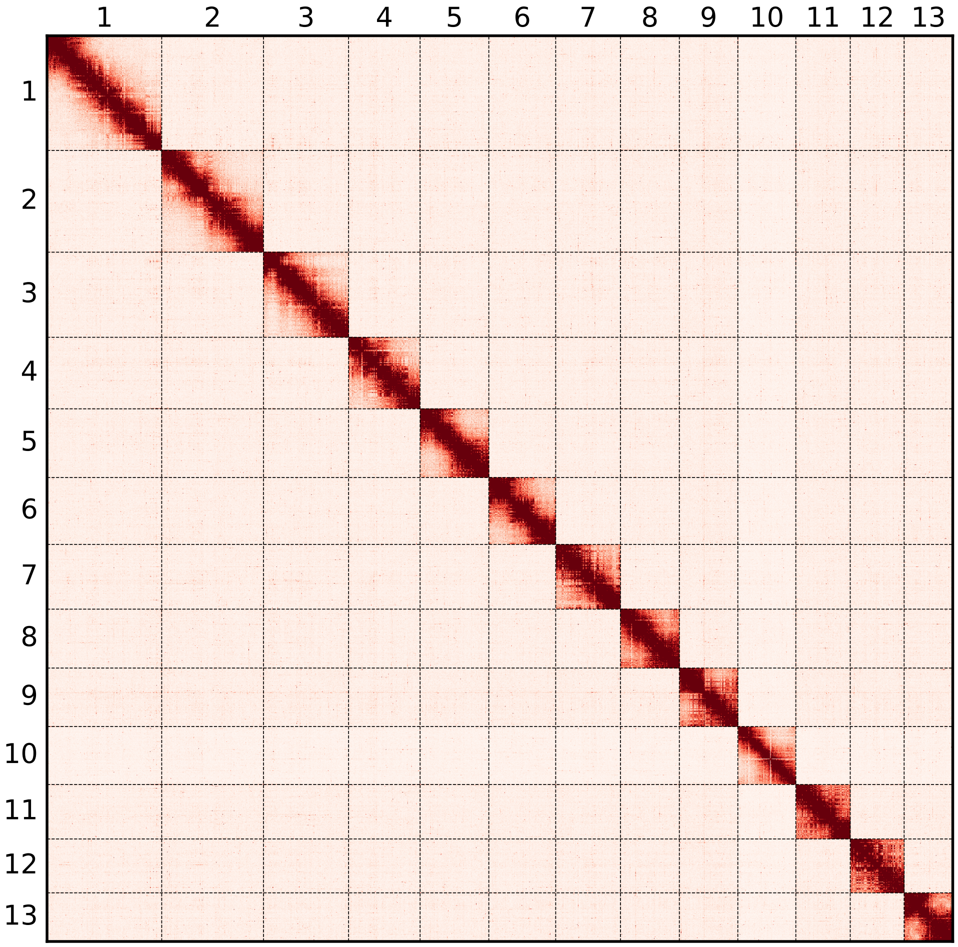


Figure S2: A HiC contact heatmap showing the 13 *Brenthis daphne* chromosomes. An analogous heatmap for *B. ino* is presented in Figure 2A of Mackintosh *et al.* (2022).

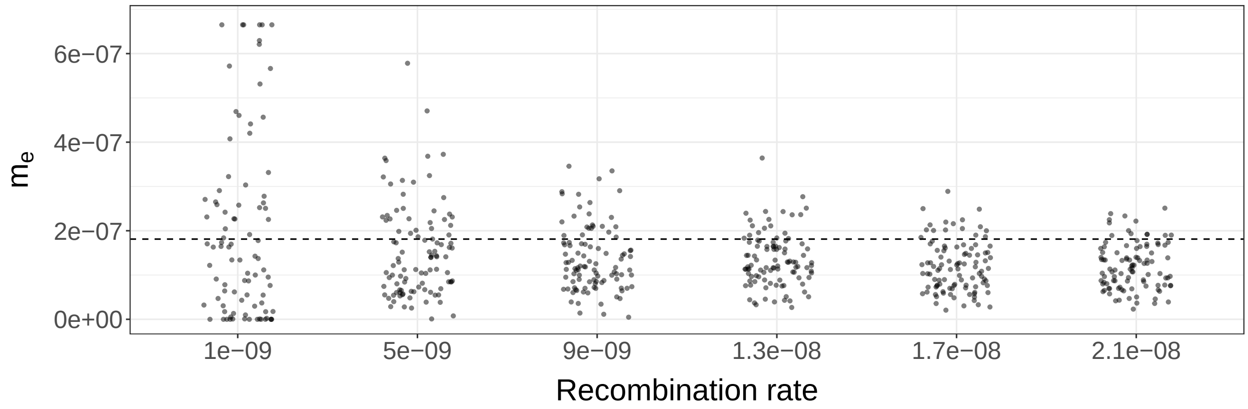


Figure S3: Estimates of effective migration rate ($m_e$) from simulations with different recombination rates. Simulation replicates are plotted as jittered points around the recombination rate that they were simulated under. The simulated $m_e$ ($1.811 \times 10^{-7}$) is plotted as a dashed horizontal line.

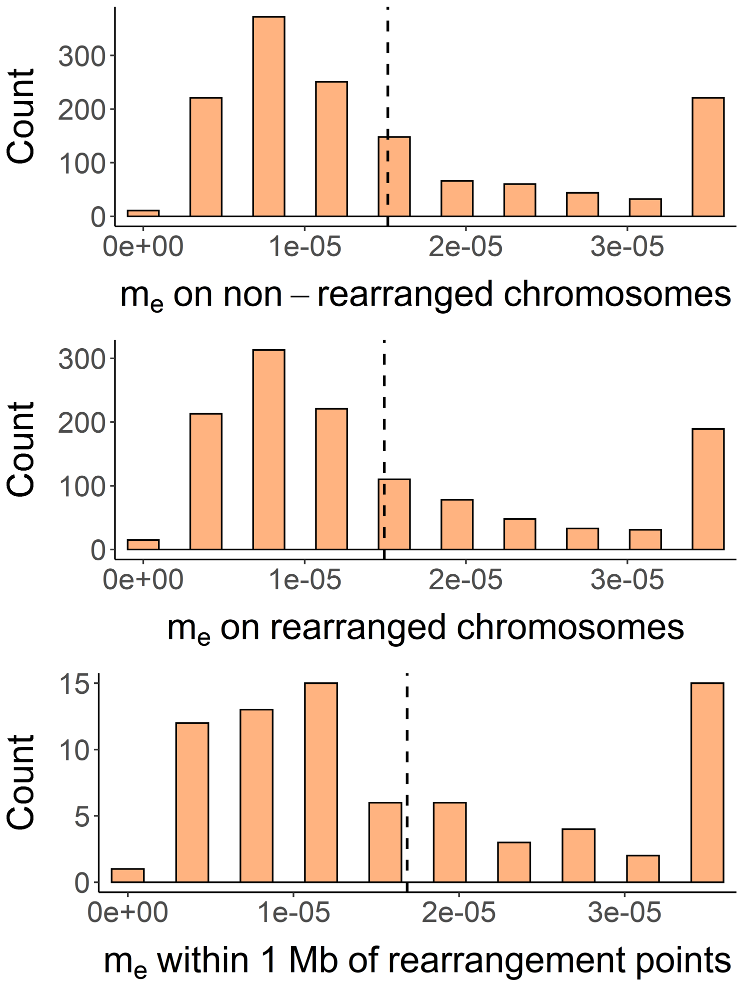


Figure S4: The distribution of $m_e$ estimates between Iberian and Balkan *B. daphne* populations for genomic windows from non-rearranged and rearranged chromosomes, as well as within 1 Mb of rearrangement points. Vertical dashed lines represent the mean of each distribution. The high frequency of windows with a maximum $m_e$ value included in the grid reflects the long tail of the $m_e$ distribution.
